# Reference genotype and exome data from an Australian Aboriginal population for health-based research

**DOI:** 10.1038/sdata.2016.23

**Published:** 2016-04-12

**Authors:** Dave Tang, Denise Anderson, Richard W. Francis, Genevieve Syn, Sarra E. Jamieson, Timo Lassmann, Jenefer M. Blackwell

**Affiliations:** 1 Telethon Kids Institute, The University of Western Australia, Subiaco, Western Australia 6008, Australia

**Keywords:** Genomics, DNA sequencing, Genetic variation, Clinical genetics

## Abstract

Genetic analyses, including genome-wide association studies and whole exome sequencing (WES), provide powerful tools for the analysis of complex and rare genetic diseases. To date there are no reference data for Aboriginal Australians to underpin the translation of health-based genomic research. Here we provide a catalogue of variants called after sequencing the exomes of 72 Aboriginal individuals to a depth of 20X coverage in ∼80% of the sequenced nucleotides. We determined 320,976 single nucleotide variants (SNVs) and 47,313 insertions/deletions using the Genome Analysis Toolkit. We had previously genotyped a subset of the Aboriginal individuals (70/72) using the Illumina Omni2.5 BeadChip platform and found ~99% concordance at overlapping sites, which suggests high quality genotyping. Finally, we compared our SNVs to six publicly available variant databases, such as dbSNP and the Exome Sequencing Project, and 70,115 of our SNVs did not overlap any of the single nucleotide polymorphic sites in all the databases. Our data set provides a useful reference point for genomic studies on Aboriginal Australians.

## Background & Summary

Whole exome sequencing (WES) is a powerful and cost-effective tool^1^ that has been employed successfully to identify causative mutations in patients affected by rare genetic diseases^2,3^. WES enables the detection of rare sequence variants specific to an individual as well as common variants that are endemic to a specific population. As there are no reference data for Aboriginal Australians to underpin the translation of health-based genomic research, we conducted a study in partnership with an Aboriginal population living at the edge of the Western Desert in Western Australia. We present a summary of the data here to be used as a reference panel to aid in the diagnosis of rare diseases and other health-based research in Aboriginal Australians.

We recently published the first Genome-Wide Association Study (GWAS) for body mass index (BMI) and type 2 diabetes (T2D) undertaken in an Aboriginal Australian population^4^. As part of the GWAS, we genotyped 402 individuals (195 of pure Martu ethnicity) using the Illumina Omni2.5 BeadChip platform. However, the design of the chip is based on single nucleotide polymorphisms (SNPs) previously identified in the HapMap^5^ and 1,000 Genomes^6^ projects, which do not include ethnically diverse groups such as the Aboriginal Australians. While the chip genotyping information is useful as a baseline to compare allele frequencies for known variants in this Aboriginal Australian sample, novel variants cannot be identified and genotyped.

**Figure 1:**
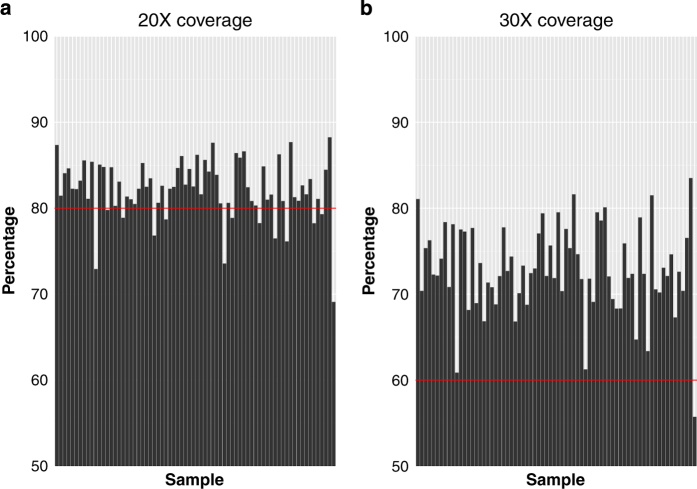
Whole exome sequencing coverage at 20X (left panel) and 30X (right panel) depth. Each bar on the x-axis represents a single sample and the percentage (starting at 50%) on the y-axis indicates the percentage of bases, out of all sequenced bases, that had at least (**a**) 20X or (**b**) 30X coverage. The red lines mark the 80 and 60% percentages.

**Figure 2:**
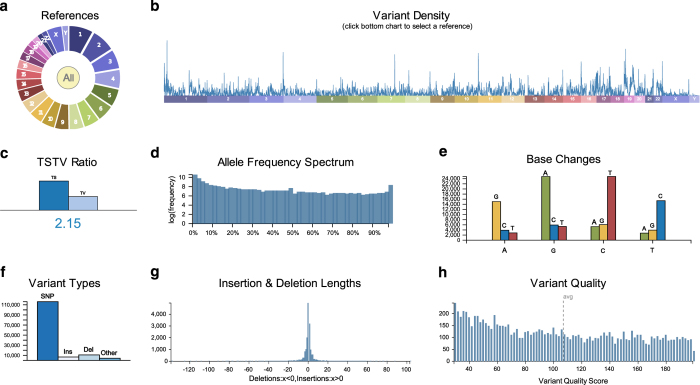
Summary metrics of Variant Call Format file generated using vcf.iobio (Miller *et al.* ^27^). Reference chromosomes are displayed in (**a**) and the distribution of variants with respect to the reference chromosomes are displayed in (**b**). The Ts/Tv ratio calculated on the sampled variants is shown in (**c**). The distribution of the alternative allele frequency (log scale) is shown in (**d**). Nucleotide substitutions per base is summarised in (**e**) and the tally of variant types is shown in (**f**). The distribution of insertion and deletion lengths is shown in (**g**) and finally the distribution of quality scores, i.e., the QUAL field, is shown in (**h**).

In continuing work, we have carried out WES on a subset of individuals (70/402) from our GWAS study and two additional individuals (a total of 72) in order to identify variants that may be associated with extreme phenotypes for BMI, renal disease, and otitis media. We processed the exome data using the Genome Analysis Toolkit (GATK) and by following their best practices guide (see Methods). Almost 80% of the sequenced bases in all the samples had a sequence depth of 20X coverage and around 60% had a sequence depth of 30X coverage (Fig. 1). Furthermore, we called a total of 320,976 single nucleotide variants (SNVs) and 47,313 insertions/deletions (indels) observed in at least one of the 72 sequenced exomes. A visual summary of a subset of the variants is shown in Fig. 2.

A comparison of our SNVs to SNPs recorded in the HapMap^5^, ESP^7^, ExAC^8^, dbSNP^9^, 1,000 Genomes^10^, and UK10K^11^ databases revealed that around 30% to 70% of our SNVs are recorded in these databases (Table 1). Our SNVs constituted the highest percentage of the ESP database (5%) (Table 1), which is expected as the ESP database contains variants determined using exome sequencing. Furthermore, 70,115 of our 320,976 SNVs (21.8%) have not been previously reported in any of these six databases. As recently reported, while most common variants are shared across the entire population, rare variants are typically restricted to closely related populations^10^. However, these unrecorded SNVs may also represent false positives, such as variants incorrectly inferred from sequencing or technical artefacts. We used the calibrated probabilities (VQSLOD) calculated using the Variant Quality Score Recalibration (VQSR) step in the GATK pipeline to gain an idea of the quality of these SNVs. Briefly, the VQSR step trains a model based on the variant annotations of known and high-quality variants in the dataset and compares unknown variants to these models, assigning a confidence score to each putative variant call. 61,212/70,115 (87.3%) of the previously unrecorded SNVs have a passing confidence score, providing confidence that these SNVs are true positives.

**Figure 3:**
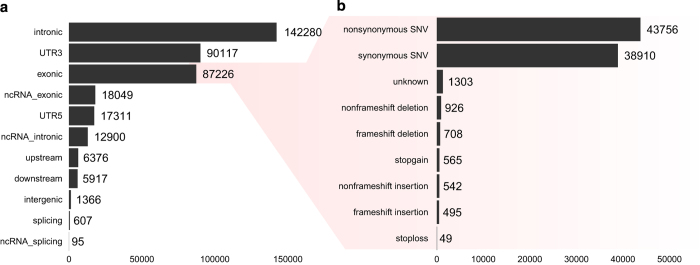
Annotation of our list of variants with respect to their genomic location (**a**) and variant consequence (**b**). Refer to the Methods for a description of the different annotation classes.

The genomic location and effect of our sequence variants with respect to protein coding genes in the RefSeq database was annotated using ANNOVAR^12^ (Fig. 3). The majority of our variants were located within introns, the 3' untranslated region (3' UTR), and exons. While the exome capture protocol mainly targets exonic regions, many of the captured DNA fragments will still fall outside of the targeted regions. We used a 100 bp padding on the targeted regions in our GATK pipeline, thereby allowing variants to be called outside of exonic regions. Intronic and 3' UTRs make up the majority of a protein coding gene, which is reflected by the number of our variants that fall within these regions. The variants that were within exonic regions were further characterised by their functional effect on protein coding genes. The majority of the sequence variants either caused a non-synonymous or synonymous mutation. While it may be expected that there should be more synonymous than non-synonymous mutations, we observed the same distribution when we annotated the UK10K and ESP6500 variants. The larger number of non-synonymous mutations is mainly caused by sequence variants that have a very low frequency in the population.

## Methods

### Study population

Subjects were recruited from an Aboriginal Australian community of Martu ancestry^13,14^ at the edge of the Western Desert in Western Australia as previously described^4^. The individuals belong to a small number of inter-related extended pedigrees and different subsets of these individuals have been diagnosed with type 2 diabetes (T2D) and/or obesity (according to their BMI).

### Chip genotyping

402 individuals were genotyped on the Illumina Omni2.5 BeadChip, which profiles 2,450,000 SNPs that were selected based on SNPs identified in the HapMap and 1,000 Genomes project. We converted the PED and MAP files into the Variant Call Format (VCF) using PLINK 1.9 (ref. 15) and hg18 coordinates were converted to hg19 coordinates using the liftOver tool^16^ and a custom Perl script (see Code availability). To ensure compatibility with the exome genotypes, we modified the REF and ALT fields (and accordingly, the genotypes) in the VCF file, such that the bases of a SNP were always on the positive strand with respect to the reference genome and the major allele was the allele present at position POS on the positive strand of the hg19 reference genome. The strandedness of the SNPs were obtained online^17^ and a custom Perl script (see Code availability) was used to modify the VCF file. The final VCF file contained genotypes for 2,380,601 SNPs due to missing calls (72 SNPs), SNPs that could not be converted to hg19 coordinates (60,142 SNPs), and missing strand information (9,185 SNPs). We did not perform any filtering of SNPs based on minor allele frequencies or call rates.

### Whole exome sequencing

We sequenced 72 samples prepared following the Illumina TruSeq protocol on an Illumina GAII outsourced to the Australian Genome Resource Facility (AGRF). The data was processed using GATK^18^ according to GATK Best Practices recommendations^19,20^ and using the GATK 2.8 data bundle^21^ for hg19; the full pipeline was implemented using Bpipe^22^ (see Code availability). Briefly, sequences were aligned to the hg19 reference genome with BWA-MEM^23^, followed by the removal of PCR duplicates, base quality score recalibration, indel realignment, and variant quality score recalibration on putative SNPs and INDELs. The VCF file produced by the pipeline uses the reference base on the positive strand of hg19 in the REF field and the variant is shown in the ALT field. We calculated the coverage using BEDTools^24^ and GNU parallel^25^ to parallelise the jobs; specifically we used bedtools coverage with the -d parameter to calculate the per base depth and then calculated the percentage of bases with at least 20X and 30X coverage.

### Overlapping with known variants

We compared our SNVs with SNPs available in six publicly available databases: HapMap 3.3 (ref. 5), dbSNP 138 (ref. 9), ESP 6500 (ref. 7), ExAC 0.3 (ref. 8), UK10K^11^, and 1KGP Phase 3 (ref. 10). We downloaded the respective VCF files and used BEDTools^24^ to intersect our SNVs to the database variants; specifically, we used bedtools intersect with the -u parameter. To obtain a list of SNVs not recorded in any of the databases, we used bedtools intersect with the -v parameter, which reports entries with no overlaps. We ran six intersects, one for each variant database, using the output from the first intersect as input to the second intersect, and so forth. Note that BEDTools only considers the genomic coordinates and doesn't take into account the alleles; hence a SNV that overlaps a SNP in a database but has different REF and ALT alleles will still count as an overlap.

### Variant annotation

We used ANNOVAR^12^ (version 2015Jun16) to annotate our genomic variants against RefSeq sequences (version 20150322), given that commercial kits target, at a minimum, all of the RefSeq genes. ANNOVAR annotates variants (in this precedence) as exonic (within coding regions), splicing (within an intron and 2 bp of a splice junction), ncRNA (within a transcript without any coding annotation), UTR5 (within a 5' untranslated region), UTR3 (within a 3' untranslated region), intronic (within an intron), upstream (within 1 kb of a transcription start site), downstream (within 1 kb of the end of a transcript), and intergenic (within an intergenic region). Variants within exons are further annotated to indicate the amino acid changes as a result of the exonic variant. Annotations include (in this precedence): frameshift insertion (an insertion leading to a frameshift), frameshift deletion (a deletion leading to a frameshift), stopgain (creation of a stop codon), stoploss (the loss of a stop codon), nonframeshift insertion (an insertion not leading to a frameshift), nonframeshift deletion (an insertion not leading to a frameshift), nonsynonymous SNV (single nucleotide change leading to an amino acid change), synonymous SNV (single nucleotide change not leading to an amino acid change), and unknown (unknown function).

### Code availability

**Figure 4:**
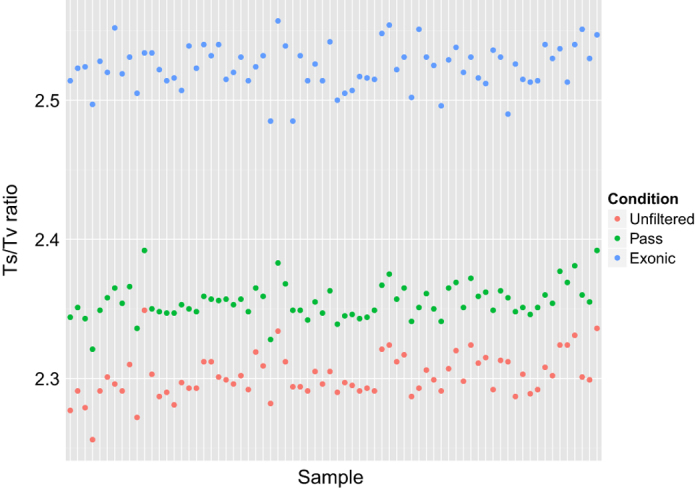
Ts/Tv ratios calculated per sample using unfiltered SNVs, SNVs passing the VQSR threshold, and SNVs passing the VQSR threshold and residing on an exonic region.

A full description of our GATK pipeline, which contains all the programs and parameters used, is openly available at https://github.com/davetang/tang_sd_2015. The Markdown file in the pipeline folder documents each step of the pipeline, as well as providing external links to the relevant sources for further information. In addition, we have provided the two Perl scripts that were used to prepare the Illumina Omni2.5 BeadChip files, and the R and TeX code used to generate Figs 1,3 and 4.

## Data Records

The unprocessed Illumina Omni2.5 BeadChip data is available from the European Genome-phenome Archive (EGA) under the accession number EGAS00001001004 (Data Citation 1) and more information on this data set can be found in our recently published study^4^. We have also deposited our full set of variants identified from our WES study as a single multi-sample VCF file in the EGA under the accession number EGAS00001001585. The raw FASTQ files are not available in the data archive, however all the steps used to process the raw files to create the final VCF file, are available at our GitHub repository (see Code availability).

## Technical Validation

70 of the 72 individuals who had their exomes sequenced were also chip genotyped, allowing us to compare the genotypes at overlapping sites. We found 83,806 comparable sites, i.e., loci where we have genotype information across the 70 individuals from both technologies, which corresponded to 5,784,674 genotypes (out of a maximum of 5,866,420 due to missing data). Using SnpSift^26^ we found that 5,701,729 of the 5,784,674 comparable genotypes were concordant (98.6%), i.e., both technologies called the same genotype (homozygous reference, heterozygous, or homozygous non-reference) at the same loci for a particular individual (Table 2). Furthermore, SnpSift only compares sites where the REF and ALT fields are identical, otherwise an error is reported; however, we only observed 178 error cases.

In addition, we calculated the transition/transversion (Ts/Tv) ratios per exome sample as a quality metric. Transitions are interchanges between purines (A, G) or pyrimidines (C, T) and transversions are interchanges between purines and pyrimidines. When there is no bias towards either transitions and transversions, the ratio is 0.5. While there are more possible transversions than transitions, transitions are observed more frequently due to molecular mechanisms and transitions are less likely to result in non-synonymous mutations; therefore we should observe a ratio greater than 0.5.

Unfiltered SNVs had the lowest Ts/Tv ratios per sample compared to SNVs that were filtered using the VQSLOD score and filtered by exonic region (Fig. 4). Due to the nature of the exome capture, regions slightly outside of the exon are captured; this is also by design to capture non-coding regions that have functional implications such as the 5′ and 3′ UTRs. However, as these sites are not as constrained (i.e. under purifying selection to maintain function) as exons, transversions are more common and can lower the Ts/Tv ratio. While it has been reported anecdotally that WES Ts/Tv ratios are around the 2.8 value, there is no general consensus. It may be possible that by using additional filters that the Ts/Tv ratio can be increased, however, we would like to present the data as is and users may apply their preferred hard filters.

## Usage Notes

Ethical approval for the study was obtained from the Western Australian Aboriginal Health Ethics Committee (Reference 227 12/12). This ethics committee is responsible for reviewing health and medical research undertaken in Western Australian Aboriginal communities. It is registered with the National Health and Medical Research Council's (NHMRC’s) Australian Health Ethics Committee (AHEC) and operates in accordance with the NHMRC National Statement on Ethical Conduct in Human Research 2007.

The data is made available through the European Genome-phenome Archive (EGA), subject to review by a study-specific Data Access Committee (DAC). Access to data will be granted to qualified researchers for appropriate health related uses. A qualified researcher refers to a scientist, who is employed, or a student enrolled at, or legitimately affiliated with an academic, non-profit or government institution, or a commercial company performing Aboriginal health related diagnostic services. Applicants are asked to complete a basic application form (which includes a brief summary of the proposal, so the DAC can determine if the planned usage falls within consents) and to agree to the terms and conditions laid out in the Data Access Agreement (DAA). The DAA must be signed by the applicant and the relevant Head of Department, Head of Institute, or equivalent. If applications include a named collaborator then the collaborator’s Institution must sign and submit a separate Data Access Agreement. Review by the DAC takes 2 weeks and if the application is approved, access via the EGA is then arranged for the applicant (https://www.ebi.ac.uk/ega/about/access). The application form, data access agreement, and further information are available from our website: http://bioinformatics.childhealthresearch.org.au/AGHS/.

Permission to lodge de-identified genotype and basic demographic data (broad geographical location, age, sex and phenotype information) in the EGA was obtained from the Board of the local Aboriginal Health Service. It should be noted that this Board approval is for use of the data in health-based research, and not for use in pure population genetics research. The data is provided as reference data for health-based research and translation in Aboriginal Australian communities.

## Additional Information

**How to cite this article:** Tang, D. *et al.* Reference genotype and exome data from an Australian Aboriginal population for health-based research. *Sci. Data* 3:160023 doi: 10.1038/sdata.2016.23 (2016).

## Supplementary Material



## Figures and Tables

**Table 1:** Overlap between the 320,976 loci detected using whole exome sequencing and SNPs recorded in six public databases.

**Database**	**Overlapping**	**Percent overlapping**	**Database size**	**Percent of database**
HapMap 3.3	97,786	30.5	3,627,692	2.7
ESP 6500	99,284	30.9	1,872,875	5.3
ExAC 0.3	130,423	40.6	8,880,581	1.47
UK10K	187,043	58.3	42,424,188	0.44
1KGP Phase 3	212,798	66.3	81,443,083	0.26
dbSNP 138	226,943	70.7	53,687,179	0.42

**Table 2:** Genotype concordance between whole exome sequencing and genotyping on the Omni2.5 platform.

**Type**	**Count**	**Percent of total**
REF/REF	3,140,230	54.3
REF/ALT1	24,011	0.42
REF/ALT2	28,140	0.49
ALT1/REF	9,275	0.16
ALT1/ALT1	1,353,334	23.4
ALT1/ALT2	3,654	0.06
ALT2/REF	12,050	0.21
ALT2/ALT1	5,815	0.1
ALT2/ALT2	1,208,165	20.9
Total	5,784,674	100
The genotypes are: homozygous reference (REF), heterozygous (ALT1), and homozygous non-reference (ALT2). The pairs of genotypes, e.g., REF/REF, on each row represent genotype calls by exome sequence and by the Omni chip respectively. REF/REF, ALT1/ALT1, and ALT2/ALT2 indicate concordant genotypes.		
